# *Notes from the Field:* Deaths Related to Hurricane Ida Reported by Media — Nine States, August 29–September 9, 2021

**DOI:** 10.15585/mmwr.mm7039a3

**Published:** 2021-10-01

**Authors:** Arianna Hanchey, Amy Schnall, Tesfaye Bayleyegn, Sumera Jiva, Anna Khan, Vivi Siegel, Renée Funk, Erik Svendsen

**Affiliations:** ^1^Division of Environmental Health Science and Practice, National Center for Environmental Health, CDC; ^2^Office of the Director, Agency for Toxic Substances and Disease Registry, Atlanta, Georgia.

On August 29, 2021, Hurricane Ida made landfall near Port Fourchon, Louisiana, as a Category 4 hurricane with sustained winds of 150 mph, causing life-threatening storm surges, wind damage, heavy rainfall, and power outages that affected approximately one million homes and businesses along the U.S. Gulf Coast (*1*,*2*). The storm then traveled Northeast as a tropical depression, causing flash flooding, tornadoes, and power outages, before exiting offshore.* During Hurricane Ida’s widespread geographic impact, collection and analysis of timely data were necessary to understand regional differences, such as causes and circumstances of death, and to guide public health messaging to promote action (*3*). In response to the disaster, CDC’s Epidemiology Surveillance Task Force^†^ (Epi/Surv Task Force) activated media mortality surveillance to track online reports of deaths related to Hurricane Ida using standardized key search terms from an internal standard operating procedure that outlines surveillance protocol. Team members compiled and coded the information from identified sources (e.g., news media articles, press releases, and social media posts) into a database, analyzed the compiled data, and shared results with emergency response leadership and health communicators to provide situational awareness and guide messaging.^§^

As of September 9, 2021, the media reported 91 deaths caused by Hurricane Ida across nine states, 56 (61.5%) of which occurred in the Northeast (Table). Among 71 (78.0%) decedents with known age, 29 (40.8%) were aged ≥65 years. By cause of death, the majority of deaths (55; 60.4%) occurred by drowning, most (52; 94.5%) of which occurred in the Northeast. Four reported deaths (4.4%) were work-related, either associated with the emergency response (three) or workplace (one). The top three circumstances of death were drowning (34; 37.4%), vehicular (22; 24.2%), and generator- or power outage–related (17; 18.7%). Cause of death is defined as the specific injury or condition that leads to death; circumstance of death is the determination of how the specific injury or condition leads to death. Among the vehicular deaths, 20 (90.9%) were drownings (e.g., submerged vehicles). The date of death was known for 60 (65.9%) reported deaths; among these, 51 (85.0%) were reported within 24 hours of the death and 34 (51.6%) were reported by media within 24 hours of regional storm impact. Hurricane Ida is the fourth most deadly hurricane the Epi/Surv Task Force has tracked in the contiguous United States since 2012; only Hurricane Harvey (2017) resulted in more reported drowning deaths (Supplementary Figure, https://stacks.cdc.gov/view/cdc/110013).

**TABLE:** Characteristics of reported deaths* related to Hurricane Ida — nine states, August 29–September 9, 2021

Characteristic	No. of deaths (%)^†^									
	Total	Louisiana	Mississippi	Alabama	New York^§^	New Jersey^§^	Pennsylvania^§^	Connecticut^§^	Virginia	Maryland
**Total**	91 (100)	28 (30.8)	2 (2.2)	2 (2.2)	18 (19.8)	32 (35.2)	5 (5.5)	1 (1.1)	1 (1.1)	2 (2.2)
**Sex**										
Female	24 (26.4)	6 (21.4)	0 (—)	0 (—)	9 (50.0)	8 (25.0)	1 (20.0)	0 (—)	0 (—)	0 (—)
Male	49 (43.8)	20 (71.4)	2 (100)	2 (100)	8 (44.4)	13 (40.6)	3 (60.0)	1 (100)	0 (—)	0 (—)
Unknown	18 (19.8)	2 (7.1)	0 (—)	0 (—)	1 (5.6)	11 (34.4)	1 (20.0)	0 (—)	1 (100)	2 (100)
**Age group, yrs**										
0−17	1 (1.1)	0 (—)	0 (—)	0 (—)	1 (5.6)	0 (—)	0 (—)	0 (—)	0 (—)	0 (—)
18−64	41 (45.1)	13 (46.4)	2 (100)	2 (100)	7 (38.9)	14 (43.8)	1 (20.0)	1 (100)	0 (—)	1 (50.0)
≥65	29 (31.9)	14 (50.0)	0 (—)	0 (—)	5 (27.8)	7 (21.9)	3 (60.0)	0 (—)	0 (—)	0 (—)
Unknown	20 (22.0)	1 (3.6)	0 (—)	0 (—)	5 (27.8)	11 (34.4)	1 (20.0)	0 (—)	1 (100)	1 (50.0)
**Work-related**										
No	51 (56.0)	25 (89.3)	0 (—)	0 (—)	12 (66.7)	9 (28.1)	3 (60.0)	0 (—)	0 (—)	2 (100)
Paid	4 (4.4)	1 (3.6)	0 (—)	2 (100)	0 (—)	0 (—)	0 (—)	1 (100)	0 (—)	0 (—)
Volunteer	0 (—)	0 (—)	0 (—)	0 (—)	0 (—)	0 (—)	0 (—)	0 (—)	0 (—)	0 (—)
Unknown	36 (39.6)	2 (7.1)	2 (100)	0 (—)	6 (33.3)	23 (71.9)	2 (40.0)	0 (—)	1 (100)	0 (—)
**Cause of death^¶^**										
Drowning	55 (60.4)	2 (7.1)	0 (—)	0 (—)	18 (100)	28 (87.5)	3 (60.0)	1 (100)	1 (100)	2 (100)
Blunt force trauma	5 (5.5)	2 (7.1)	2 (100)	0 (—)	0 (—)	0 (—)	1 (20.0)	0 (—)	0 (—)	0 (—)
CO poisoning	6 (6.6)	6 (21.4)	0 (—)	0 (—)	0 (—)	0 (—)	0 (—)	0 (—)	0 (—)	0 (—)
Electrocution	3 (3.3)	0 (—)	0 (—)	2 (100)	0 (—)	1 (3.1)	0 (—)	0 (—)	0 (—)	0 (—)
Preexisting condition	6 (6.6)	6 (21.4)	0 (—)	0 (—)	0 (—)	0 (—)	0 (—)	0 (—)	0 (—)	0 (—)
Hyperthermia	10 (11.0)	10 (35.7)	0 (—)	0 (—)	0 (—)	0 (—)	0 (—)	0 (—)	0 (—)	0 (—)
Other/Unknown**	6 (6.6)	2 (7.1)	0 (—)	0 (—)	0 (—)	3 (9.4)	1 (20.0)	0 (—)	0 (—)	0 (—)
**Circumstance of death^††^**										
Drowning	34 (37.4)	1 (3.6)	0 (—)	0 (—)	14 (77.8)	15 (46.9)	1 (20.0)	0 (—)	1 (100)	2 (100)
Trauma (tree or building)	1 (1.1)	1 (3.6)	0 (—)	0 (—)	0 (—)	0 (—)	0 (—)	0 (—)	0 (—)	0 (—)
Generator/Power outage	17 (18.7)	17 (60.7)	0 (—)	0 (—)	0 (—)	0 (—)	0 (—)	0 (—)	0 (—)	0 (—)
Vehicular	22 (24.2)	1 (4.5)	2 (100)	0 (—)	4 (22.2)	12 (37.5)	2 (40.0)	1 (100)	0 (—)	0 (—)
Preparedness/Repair injury	5 (5.5)	1 (3.6)	0 (—)	2 (100)	0 (—)	2 (6.3)	0 (—)	0 (—)	0 (—)	0 (—)
Other	7 (7.7)	6 (21.4)	0 (—)	0 (—)	0 (—)	0 (—)	1 (20.0)	0 (—)	0 (—)	0 (—)
Unknown	5 (5.5)	1 (3.6)	0 (—)	0 (—)	0 (—)	3 (9.4)	1 (20.0)	0 (—)	0 (—)	0 (—)

**Abbreviation**: CO = carbon monoxide.

* The Epidemiology Surveillance Task Force scans media reports daily for confirmed and unconfirmed deaths using key search terms according to a standard operating procedure. https://www.cdc.gov/nceh/hsb/disaster/surveillance.htm

^†^ Percentages might not sum to 100% because of rounding.

^§^ States in the Northeast that were affected by Hurricane Ida.

^¶^ Cause of death is the specific injury or condition that leads to death.

** Other/unknown includes alligator attack in floodwater and unknown cause of deaths (e.g., insufficient information at the time).

**^††^** Circumstance of death is the determination of how the specific injury or condition leads to death.

The type of surveillance described in this report can help reveal the diversity in outcomes from the same type of incident and allows CDC to respond quickly to specific public health threats. For example, during Hurricane Laura (2020), messaging focused on carbon monoxide exposures; during Hurricane Florence (2018), the primary concern was driving through floodwaters. During Hurricane Ida, the most recently reported deaths were discovered during wellness checks; therefore, messaging focused on checking on loved ones. Such evidence-based messaging, delivered through multiple channels to reach diverse audiences, is critical to saving lives, minimizing injury, and protecting public health. Leveraging the work of reporters on the ground who provide information about the current situation is important to this effort and facilitates the tracking of circumstances of death and helps target risk communication and messaging.

The findings in this report are subject to at least one limitation. Media reports are not official records and might not reflect all disaster-related deaths. CDC’s Epi/Surv Task Force will continue to work with partners to help improve the accuracy and timeliness of official mortality data sources.

The media represent an immediate resource for timely information during an emergency response (*4*). CDC’s Epi/Surv Task Force uses media reports of both confirmed and unconfirmed deaths to guide evidence-based public health messaging to help prevent further injury and death.^¶^ For example, reports of motor-vehicle involved drownings, whether confirmed or not, can help guide geographic targeting and timing for phase-based messages, such as avoiding driving in floodwaters, and can support existing coordination with state and local communicators (*5*). Continued use of media reports of both confirmed and unconfirmed deaths can guide evidence-based public health messaging to help prevent further injury and death.

## Footnotes

*
https://www.nhc.noaa.gov/archive/2021/al09/al092021.discus.015.shtml

† This is the formal name for the current Incident Management System for Hurricane Ida as well as previously activated CDC Emergency Operations Center hurricane response surveillance systems. https://www.cdc.gov/nceh/hsb/disaster/surveillance.htm

§
https://www.cdc.gov/disasters/hurricanes/index.html

¶ For emergency response purposes, media reports of deaths show what potential hazards and dangerous behaviors are occurring, which is vital for CDC’s Epi/Surv Task Force’s response-related public health messaging.
